# Research on the Thermal Behaviour of a Selectively Laser Melted Aluminium Alloy: Simulation and Experiment

**DOI:** 10.3390/ma11071172

**Published:** 2018-07-09

**Authors:** Zhonghua Li, Bao-Qiang Li, Peikang Bai, Bin Liu, Yu Wang

**Affiliations:** 1 School of Mechanical Engineering, North University of China, Taiyuan 030051, China; lzh2017@nuc.edu.cn; 2 School of Materials Science and Engineering, North University of China, Taiyuan 030051, China; lbqlalala@gmail.com (B.-Q.L.); liubin3y@nuc.edu.cn (B.L.); wangyu@nuc.edu.cn (Y.W.)

**Keywords:** selective laser melting, numerical analysis, thermal behaviour, AlSi10Mg alloy

## Abstract

A 3D Finite Element (FE) model was developed to investigate the thermal behaviour within the melt pool during point exposure to Selective Laser Melting (SLM) processed AlSi10Mg powder. The powder–solid transition, temperature-dependent thermal properties, melt pool convection, and recoating phase were taken into account. The effects of Exposure Time (ET) and Point Distance (PD) on SLM thermal behaviour were also investigated and showed that the short liquid phase time and high cooling rate of the melt pool reduced the viscosity of the melt pool at a lower ET or a higher PD. This resulted in poor wettability and the occurrence of balling and micropores. At a higher ET or lower PD the melt pool became unstable and allowed for easy formation of the self-balling phenomenon, as well as further partial remelting in the depth direction resulting in the creation of larger pores. The proper melt pool width (119.8 μm) and depth (48.65 μm) were obtained for a successful SLM process using an ET of 140 μs and a PD of 80 μm. The surface morphologies and microstructures were experimentally obtained using the corresponding processing conditions, and the results aligned with those predicted in the simulation.

## 1. Introduction

Al-Si alloys have excellent properties such as low density, specific strength, stiffness, and electrical and thermal conductivity. Due to this, they have become one of the desirable lightweight materials in aerospace, automotive, microelectronics, and other mechanical instrument industries. The hypoeutectic alloy is close to the eutectic composition (12.5% Si) and therefore has excellent castability and weldability in comparison to other Al alloys. Hypoeutectic alloy AlSi10Mg is a fitting example in this regard. Owing to the combination of its light weight and good mechanical properties, this alloy has been widely used for satellites, space stations, and other space vehicles. Precipitation hardening also plays an important role in its performance, as it enables precipitating Mg_2_Si during age hardening [1]. However, it is difficult to manufacture complex integrated components through conventional manufacturing technologies [2]. This restricts further development and applications of AlSi10Mg. Therefore, new processing methods are in high demand.

Selective Laser melting (SLM) is an emerging additive manufacturing process that provides substantial flexibility in the production of components with complex shapes [3]. In SLM, there are two different laser scan patterns: point exposure scan and continuous exposure scan. Both processes of the metallic powders are based on a complete melting/solidification mechanism, which indicates suitability in producing fully dense components approaching 99.9% relative density [4]. Moreover, the technology can produce near-net-shaped parts with customised and complicated structures in a direct way; it is extremely suitable for manufacturing the final part with almost complete geometric freedom. Despite its great economic and technological advantages, the technology still has some drawbacks in terms of processing stability, processing accuracy, and surface quality. The quality of the formed part largely depends on the stability of the melting process [5,6]. The temperature and stability of the melt pool play a crucial role in the SLM process, which directly affects the shape of the melt pool and the mechanical properties of the final parts. At present, part quality is determined mainly by optimising parameters (such as laser power and scanning speed). As trial and error requires a long period and is costly, the numerical simulation method is preferred to process optimisation [7]. Combining the predicted results of numerical simulation with a small amount of experimental optimisation will significantly reduce the cycle of production and costs.

There has been some recent valuable research aimed at simulating the SLM process. Li et al. [8] established a Finite Element (FE) model and studied the temperature fields during the SLM process with AlSi10Mg powder under different laser powers and scanning speeds. The results indicated that relatively higher scanning speeds produced a narrower melt pool width, causing large gaps and pores along the scanning direction. Following on, Loh et al. [9] proposed a FE model to achieve volume shrinkage and vaporisation in SLM-processed AA6061 parts. The results showed that laser power had a significant influence on the evaporation of molten materials because the heat in the centre of the laser beam could not spread out quickly. Yu et al. [4] established a 3D mesoscopic model using the Finite Volume Method so as to investigate the thermal behaviour of the melt pool during the SLM of AlSi10Mg powder under different laser powers and scanning speeds. It was found that both low and high laser power led to a poor surface quality. High volume energy density caused the balling phenomenon due to excessive liquid formation. Along similar lines, Han et al. [10] developed a FE model and simulated the transient temperature distribution and configurations of the melt pool during the SLM of Al–Al_2_O_3_ composite powder by changing the hatch spacing and scanning speed. The maximum temperature showed a slight increase along the scanning route; this trend was more obvious at low scanning speeds.

The abovementioned investigations into SLM-processed Al alloy powder are all focused on thermal behaviours caused by parameters which are all in respect of the laser scan pattern of the continuous exposure scan. Limited research exists into the point exposure process and defect prediction. During the continuous exposure scan process, a continuous laser travels over the surface of the powder with a constant scanning speed. While in the point exposure scan process, the material is heated intermittently with a succession of short-duration pulses, the laser moves step by step with a specific Exposure Time (ET) and Point Distance (PD). In general, the scanning speed is determined by the PD and ET in some literature [2,11]. In actual fact, the pulsed laser can easily melt powder with higher peak temperature. This kind of SLM process offers the advantage of very low heat input to the melt pool, which helps to deposit temperature-sensitive components with low distortion.

In this paper, a 3D FE model was introduced to simulate the thermal behaviour and melt pool dimensions of AlSi10Mg alloy parts in the process of point exposure scan and to predict the relationship between processing parameters and microstructure defects. Parametric analysis was developed under varying ET and PD. Moreover, corresponding experiments were carried out to study the microstructure of components produced by SLM, in order to verify the numerical results.

## 2. Model for SLM Process

### 2.1. Mathematical Modelling of Heat Transfer

**Figure 1:**
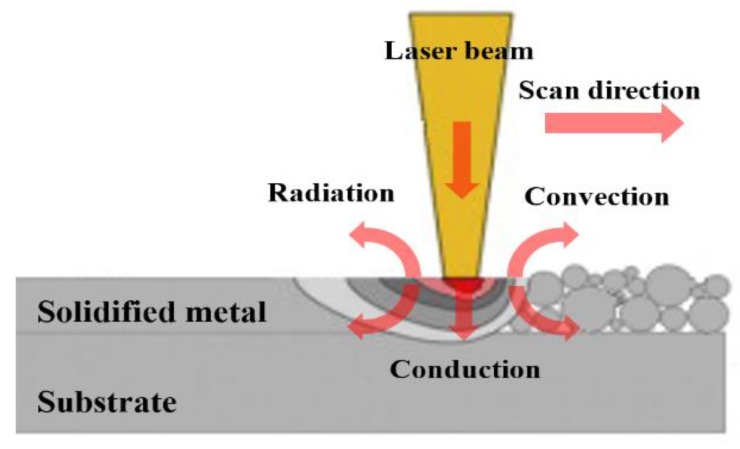
Schematic of thermal behaviour of powder bed under laser irradiation.

Figure 1 depicts the schematic diagram of the SLM process. During the scan, the laser beam is irradiated on the top surface of the powder bed. Here, a series of complicated physical and chemical phenomena occur in the interaction process of the laser and powder materials, which includes two phases: (1) the reflectivity and absorption of the material’s surface to the laser and (2) heat transfer between powder particles, the processed part and the surrounding environment [8].

The powder is firstly melted by the laser beam, following which the metallurgic bonding is formed. In the process of heat transfer, it is assumed that the powder bed and its surrounding environment constitute a closed and insulated system. The energy balance follows the first law of thermodynamics, which includes thermal conduction, and the heat losses due to convection and radiation. In Cartesian coordinates, the spatial and temporal distribution of the temperature field of *D* can be represented by a three-dimensional heat transfer differential equation, which can be expressed as [12]:

(1) $$\rho c\frac{\partial T}{\partial t}=k_{p}\left(\frac{\partial^{2}T}{\partial x^{2}}+\frac{\partial^{2}T}{\partial y^{2}}+\frac{\partial^{2}T}{\partial z^{2}}\right)+Q,\;\left(x,y,z\in D\right)$$

where ρ is the material density (kg/m^3^); c is the specific heat capacity (J/(kg·°C)); T is the temperature (°C); *t* is the interaction time (s); $k_{p}$ is the effective thermal conductivity of the powder bed; and *Q* is the heat generated per volume within the component (W/m^3^).

Before the SLM process begins, the initial temperature, $T_{0}$, in the powder bed and substrate is equal to 25 and 80 °C respectively. The heat relationship between them follows the formula below:

(2) $$k_{p}\frac{\partial T}{\partial n}=Q+Q_{c}+Q_{r},\;\left(x,y,z\in S\right)$$

where S denotes the surfaces attached to the applied heat flux, convection and radiation; n is the normal vector of the powder surface; Q is the input heat flux from the laser, which is described specifically in the following section; and $Q_{c}$ is the heat convection and can be expressed as:

(3) $$Q_{c}=h\left(T_{s}-T_{m}\right)$$

$Q_{r}$ is the heat radiation and can be defined by:

(4) $$Q_{r}=\sigma \varepsilon \left(T_{s}^{4}-T_{m}^{4}\right)$$

Since the heat radiation process is a highly non-linear process, Equations (3) and (4) can be substituted into Equation (2). The following equation is thus derived:

(5) $$k_{p}\frac{\partial T}{\partial n}=H\left(T_{s}-T_{m}\right),\;\left(x,y,z\in S\right)$$

In Equations (3)–(5), h is the coefficient for heat convection; σ is the Stefan–Boltzmann constant; ε is the emissivity and equal to 0.3 [9]; $T_{s}$ is the model surface temperature; $T_{m}$ is the ambient temperature (25 °C); and *H* is the effective heat transfer coefficient.

### 2.2. Numerical Model Establishment

**Figure 2:**
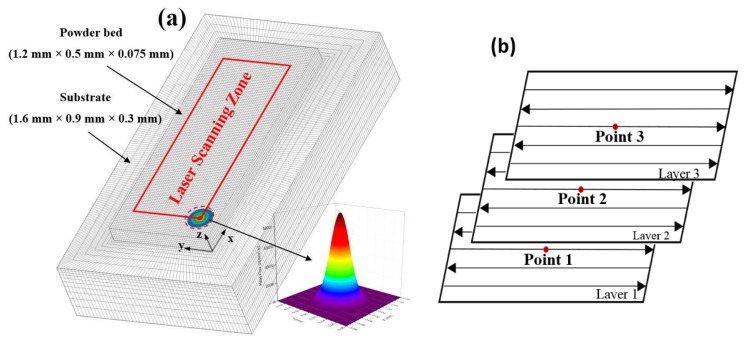
3D Finite Element (FE) model and Gaussian laser energy density (**a**) and laser scanning pattern in Selective Laser Melting (SLM) process (**b**). (Point 1 at the centre of layer 1, Point 2 at the centre of layer 2 and Point 3 at the centre of layer 3 respectively).

In order to align with the real operating conditions for the SLM process of AlSi10Mg, a 3D transient numerical model was developed using ANSYS 16.0 commercial FEM software. The FE model and the laser scanning pattern in the SLM process are shown in Figure 2. The model in Figure 2a includes an ENAW-5083 Al alloy substrate positioned underneath and an upside powder bed with dimensions of 1.6 mm × 0.9 mm × 0.3 mm and 1.2 mm × 0.5 mm × 0.075 mm, respectively. Figure 2b shows the movement of the laser in a reciprocating raster pattern. In order to improve the calculation accuracy of the model, the SOLID 70 hexahedron elements with a fine mesh of 0.01 mm × 0.01 mm × 0.0125 mm were used in the powder bed with a layer thickness 0.025 mm.

**Figure 3:**
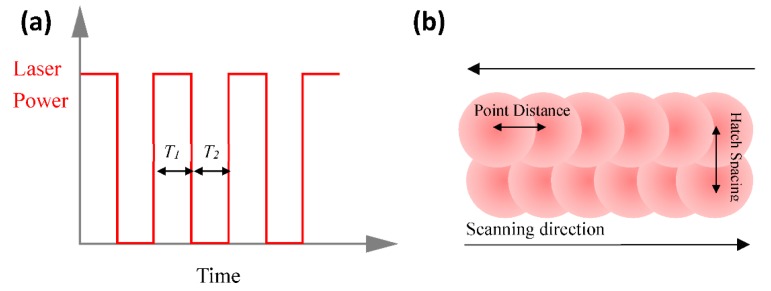
The waveform of the modulated pulsed laser power output (**a**) and the laser working mode (**b**).

The laser beam had a spot size of 80 μm and was treated with a surface heat flux boundary condition in analysis; its energy density nearly followed a Gaussian function as shown in Figure 2a. The laser moved step by step with the specific ET and PD in the X-direction on the powder bed (named the modulated pulsed laser by Renishaw PLC, Wotton-under-Edge, UK). Parameter ET, represented by T_1_, concerns the time for which each point is radiated by the laser before jumping to the next point, while the PD data indicates the distance between the adjacent points. The schematic diagram is shown in Figure 3. At a fixed PD value equal to 80 μm, the varying ET values were applied from 100 μs to 180 μs in increments of 20 μs. At a fixed ET value equal to 140 μs, the PD values varied from 60 μm to 100 μm in increments of 10 μm. The process parameters used in the study are shown in Table 1.

### 2.3. Modelling of Laser Energy

During the SLM process, the material was molten through a laser beam. The laser intensity distribution almost followed a Gaussian relationship, which is mathematically expressed as:

(6) $$Q\left(r\right)=\frac{2AP}{\pi R^{2}}exp\left(-\frac{2r^{2}}{R^{2}}\right)$$

where P is the laser power; r is the real-time distance to the centre of the laser spot; *R* is the effective radius of the heat source on the material surface where the energy density is reduced to 1/e^2^ at the centre of the laser spot; and *A* is the laser energy absorptivity of the material. The absorptivity of fibre laser radiation by a polished AlSi10Mg surface is around 9% at room temperature. However, the laser absorptivity of a powder material is 2–3 times higher than the counterpart block material. An absorptivity level of 12% for material AlSi10Mg is used by Ding et al. [14]. In the simulation, an absorptivity of 18% was used, as per Wei et al. [13]

During the SLM process, the latent heat of the phase change must not be ignored, and is usually treated by enthalpy, *H* (J/m^3^), which can be written as a function of temperature, *T*, and the specific heat, C:

(7) $$H=\int_{T_{ref}}^{T}\rho CdT$$

### 2.4. Thermal Physical Parameters

Temperature has a fundamental influence on the microscopic structures and mechanical properties formed during the radiation of the AlSi10Mg alloy powders by the laser in the SLM processing. The density, specific heat capacity, and thermal conductivity are indispensable for the purpose of obtaining the temperature field and the effective thermal conductivity of powders determines the validity of the simulation of SLM among them.

**Figure 4:**
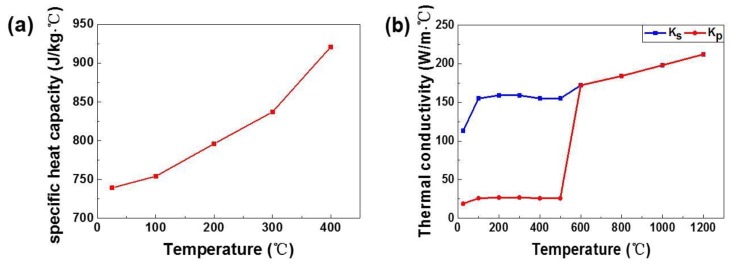
Temperature-dependent thermal properties: specific heat capacity (**a**) and the effective thermal conductivity of AlSi10Mg powder and solid (**b**).

The temperature-dependent thermophysical parameters of AlSi10Mg are shown in Figure 4 [15]. The twofold thermal conductivity is used to account for melt pool convection beyond the melting temperature [16]. The effective thermal conductivity of powder $k_{p}$ can be estimated as follows:

(8) $$k_{p}=k_{s}\frac{pn}{\pi }x$$

where $k_{s}$ is the thermal conductivity of solid AlSi10Mg; p is the relative density of the powder bed assigned at 0.6; n is the coordination number equal to 6; and x is the contact size ratio.

## 3. Experiments

In this study, an AlSi10Mg powder with an average particle size of 45 μm, supplied by LPW Technology (LPW Technology Ltd., Widnes, Cheshire, UK) was used. A Renishaw AM 400 SLM system (Renishaw PLC, Wotton-under-Edge, UK) that employs a modulated ytterbium fibre laser with a wavelength of 1070 nm was utilised to fabricate cubic samples in order to verify the results of the simulation. The entire SLM process was also conducted in an argon atmosphere. The spot size of the laser beam focused on the powder bed was 80 μm. The process parameters employed were the same as those in the simulations (Table 1). Samples for metallographic examinations were cut, ground, and polished according to standard procedures, and then etched with Keller’s reagent for 10 s. Mechanical polishing was adopted by means of a high-speed polishing round and the samples were polished up to 1.5 μm using diamond polishing paste. A ZEISS Scope. A1 OM microscope (Zeiss, Jena, Germany) was used to observe the low-magnification cross-sectional microstructures of specimens. The OM images were captured from the central area of the observation plane and magnified 100 times. The characteristic top surface morphologies of the SLM-fabricated AlSi10Mg parts were achieved using a Hitachi SU-5000 scanning electron microscopy (SU-5000 SEM, Hitachi Ltd., Chiyoda-ku, Tokyo, Japan) device in secondary electron mode at 20 kV.

## 4. Results and Discussion

### 4.1. Temperature Distribution

**Figure 5:**
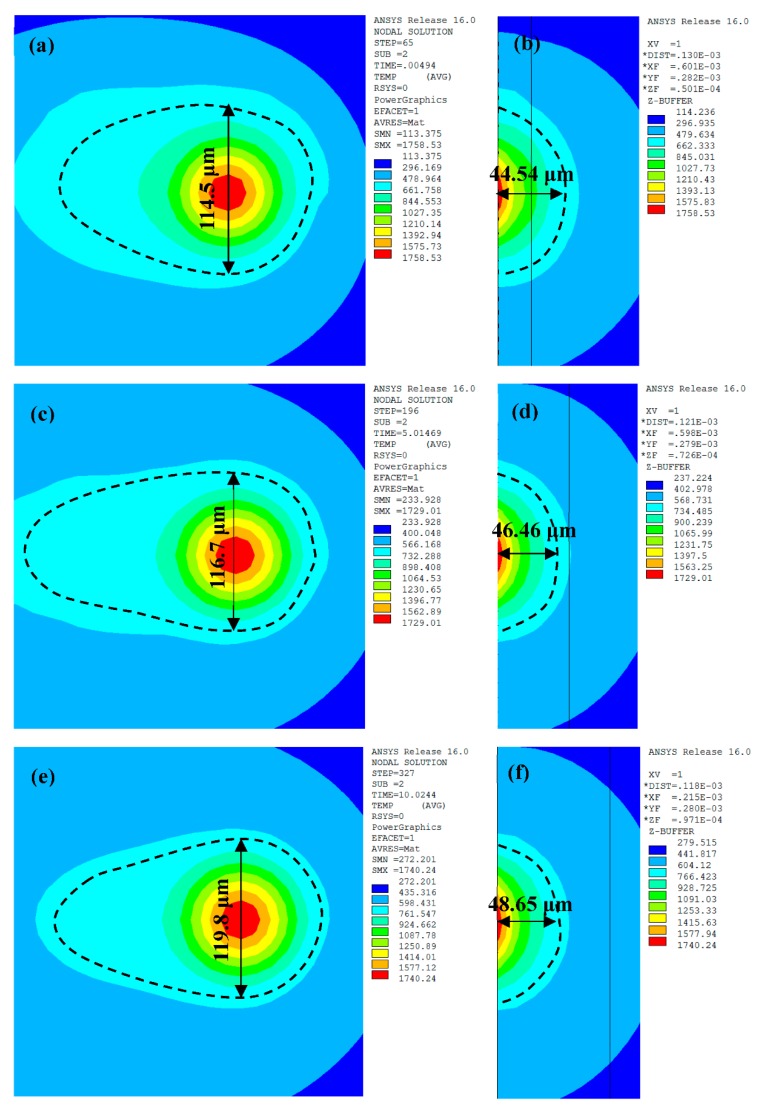
Temperature distributions of melt pool at Point 1 (**a**,**b**); Point 2 (**c**,**d**); and Point 3 (**e**,**f**) during SLM process at Exposure Time (ET) = 140 μs and Point Distance (PD) = 80 μm. The left is the top surface and the right is the cross-section.

Figure 5 shows the transient temperature distribution of the top surface and the cross section of the melt pool at the centre of the different layers during the SLM process, using ET values of 140 μs and PD of 80 μm. We can see that the melt pool had an elliptical shape, was more intensive at the forepart of the ellipses, and also included a larger temperature gradient. This was primarily due to the thermal conductivity of the powder being less than that of the solid and heat loss which occurred mainly during solidification. The dotted black line represents the isotherm of the melting temperature of AlSi10Mg (600 °C), and the temperature gradient inside of the dotted lines area is larger than that in the surrounding area. This resulted in a fine cellular structure inside of the melt pool and a heat-affected zone around the melt pool was formed during the SLM process [17].

The predicted characteristics of the melt pool varied according to the mobile laser exposure. With the laser exposed at point 1, the maximum temperature reached 1758.53 °C with a width and depth of 114.5 μm and 44.54 μm respectively (Figure 5a,b). When the laser moved to point 2, the maximum temperature of the melt pool decreased to 1729.01 °C, while the width and depth increased to 116.7 μm and 46.46 μm, respectively (Figure 5c,d). However, as the laser reached point 3, the maximum temperature increased slightly to 1740.24 °C, with the width (119.8 μm) and depth (48.65 μm) increasing by 2.67% and 4.71%, respectively (Figure 5e,f). It was clear that, although the recoating phase provided sufficient cooling time, there was still a slight heat accumulation effect during the SLM process, thus leading to an increase in the melt pool dimensions. Meanwhile, the dimensions of the melt pool were larger than those in the continuous exposure scan. Ding et al. [14] explained that there are two vortexes in the velocity field, thus leading to intense convective motion. Moreover, the range in depth of the melt pool was greater than its range in width. This was because the thermal conductivity was higher in the previous layer than that of the adjacent tracks, and more heat was conducted in the depth direction.

### 4.2. Thermal Behaviours

**Figure 6:**
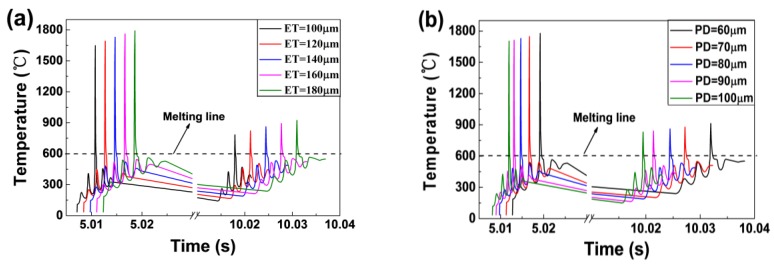
The temperature variation with time at point 2. Using different ET (PD = 80 μm) (**a**) and different PD (ET = 140 μs) (**b**).

Figure 6 shows the temperature variation with time at point 2 using different ET and PD values. The slope of the curve represents its cooling rate. As the ET increased from 100 μs to 180 μs, the cooling rate of the melt pool decreased from 7.93 × 10^6^ °C/s to 3.61 × 10^6^ °C/s. When increasing the PD from 60 μm to 100 μm the cooling rate rose significantly from 3.25 × 10^6^ °C /s to 7.48 × 10^6^ °C/s. Based on these results, it is easy to conclude that the cooling rate is more sensitive to the PD value than to that of the ET. By comparing this data with relevant literature on AlSi10Mg [8,18,19], which is built through the continuous exposure SLM, it is found that the higher cooling rate is in the point exposure SLM process. This contributes to obtaining fine grain. The temperature variation showed an apparent fluctuation behaviour and each peak represented the laser beam passing through. Each fluctuation is a scanning cycle of the track. The breaks on the x-axis represent the recoating phase in SLM (the recoating time is equal to 5 s). It is noted that the cooling rate in this phase rose with increasing ET and decreasing PD values. The differences between the cooling rates of the melt pool were due to an increased heat accumulation in the underlying solid material; therefore, more time is required to cool down. When the laser arrived at point 2, the temperature of the third peak is the highest. The powder in the area directly under the laser melted in a shorter time, helping towards fine-grain strengthening [20,21]. With the laser leaving, the region experienced a rapid fall in temperature and underwent solidification. After the third powder was spread, the region experienced a similar variation from the first track to the fifth track as the laser scanned in the same way. When the laser reached the centre of layer 3 (point 3), the temperature of point 2 was over the melting line, thus indicating that the remelting phenomenon occurred in layer 2 and metallurgical bonding took place between the adjacent layers.

**Figure 7:**
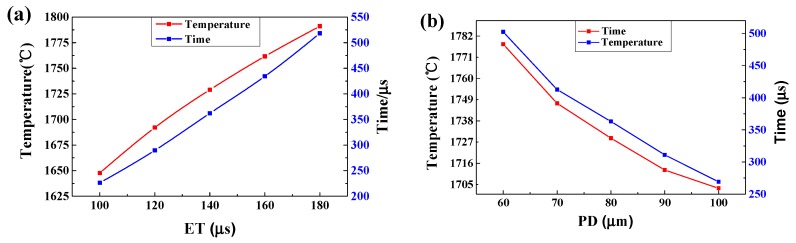
The maximum temperature and the liquid lifetime of the melt pool at point 2. Using different ET (PD = 80 μm) (**a**) and different PD (ET = 140 μs) (**b**).

Figure 7 shows the maximum temperature and the liquid lifetime at point 2 with the varying process. It can clearly be seen that the maximum temperature increased from 1692.06 °C to 1761.64 °C when the ET increased from 120 μs to 160 μs. Meanwhile, the liquid lifetime also increased from 287.56 μs to 424.28 μs, rising by 4.11% and 47.54%, respectively (Figure 7a). At the same time, as the PD increased from 60 μm to 100 μm, the maximum temperature decreased from 1777.75 °C to 1703.02 °C, decreasing by 4.20%; moreover, the liquid lifetime shortened from 502.41 μs to 269.15 μs, decreasing by 46.43% (Figure 7b). As noticed, the PD had a more pronounced impact on the liquid lifetime under the same amplitudes of variation, and this finding has a primary direct meaning for process optimisation.

In the SLM process, the maximum temperature, the cooling rate and the liquid lifetime of the melt pool depend on the applied ET and PD, which determine the formation of a stable melt pool and good metallurgical bonding. Poor melt pool configurations can reduce the density of parts and can even mean that the forming does not finish. It is therefore vital to ascertain the appropriate ET and PD in order to achieve a stable melt pool and smooth overlap.

### 4.3. Melt Pool Configurations

**Figure 8:**
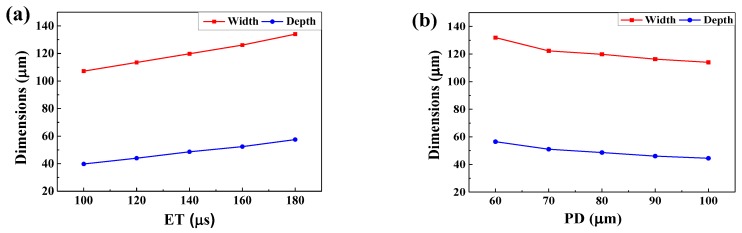
The width and depth of melt pool with different ET (PD = 80 μs) (**a**) and different PD (ET = 140 μs) (**b**).

**Figure 9:**
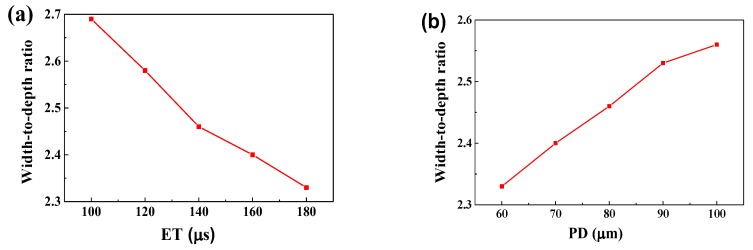
The ratio of width to depth of the melt pool with different ET (PD = 80 μm) (**a**) and different PD (ET = 140 μs) (**b**).

The melt pool shape in layer 3 has reached a steady state (Figure 5). Figure 8 shows temperature variation with different ET and PD values when the laser moved to point 3. The temperature gradients at the centre of the melt pool were much larger than those in the edge area. To improve the precision of the data, the data points with spacing of 0.5 μm were acquired using linear interpolation via the ANSYS software. As the ET increased from 100 μs to 180 μs, the width and depth increased from 107.2 μm to 134 μm and from 39.8 μm to 57.5 μm, increasing by 25% and 44.47%, respectively. The corresponding width-to-depth ratio decreased from 2.69 to 2.33, dropping by 13.38% (Figure 9a). As the PD increased from 60 μm to 100 μm, the width and depth decreased from 131.9 μm to 114 μm and from 56.5 μm to 44.5 μm, decreasing by 13.57% and 21.24%, respectively. The corresponding width-to-depth ratio increased from 2.33 to 2.56, rising by 9.87% (Figure 9b). It can be seen that both ET and PD had a more notable effect in the depth direction, which was due to the solidified layers with higher heat conductivity. Besides this, the width and depth were more sensitive to the PD than to the ET. These results were also found by Ding et al. [7]. The aforementioned authors postulated that the overall temperature around the portion of laser scanning is elevated when a larger PD is applied, which supports heat transfer in the horizontal direction. Moreover, in their study, it was found that, with regard to the ET, changing the ET from 20 μm to 280 μm had a negligible effect on heat transfer. Li et al. [8] concluded that higher laser power will penetrate deeper in the SLM process of AlSi10Mg and the morphology of melt pool is more sensitive to laser powder compared with scanning speed. The same was concluded by Zhang et al. [22]. Along similar lines, Cherry et al. [11] studied the effect of ET and PD on the quality of 316L stainless steel. They found that ET had little effect on roughness, whilst variation in the PD caused a large change in roughness.

As the ET increased and the PD reduced, except for the enlarged melt pool with the increasing laser energy density, the width-to-depth ratio was also enhanced. The same trend in the length-to-depth ratio was obtained by elevating the laser power and reducing the scanning speed in a continuous exposure scan pattern [4,8]. This is because that the melt pool overlap conditions transformed from the conduct mode to the keyhole mode. Under such circumstances, the greater the amount of the molten powder that evaporated, the deeper the melt pool became, and this resulted in a decreasing width-to-depth ratio. Stable melt pool dimensions are vital for forming an appropriate overlap between tracks. When the overlapping ratio is around 30% it ensures strong overlapping between melt pools and helps to obtain a smooth surface. In this study, the overlapping ratio was approximately 31.25% when the ET and PD were assigned to 140 μs and 80 μm, respectively. The corresponding laser energy density was 175 J/mm^3^, which is less than the optimised value (250 J/mm^3^) in the continuous exposure scan in Yu et al.’s works [8]. This is attributed to the fact that the pulsed laser has a higher peak temperature and, thus, can more easily melt powder in low energy density. However, the predicted results are in accordance with the optimal process of the point exposure scan SLM conducted by Wang et al. [2] and Aboulkhair et al. [23].

Good metallurgic bonding between melt pools is vital when it comes to decreasing the pores in the workpiece and to improving processing quality [24,25]. Therefore, under good overlapping conditions, a larger melt pool is important in order to obtain highly dense products. However, when the size of the melt pool exceeds the threshold level the melt pool becomes unstable, thus causing micropores, holes, cracks and so on. Due to the excessive energy density, a high temperature gradient is formed and causes the large thermal stress in the melt pool. At the initial position of the scan track, the cooling rate and the solidification rate remain high because of the low overall temperature. A hot crack easily leads to warpage, which has been verified for SLM-processed iron powder by Li et al. [26].

### 4.4. Experimental Investigation

**Figure 10:**
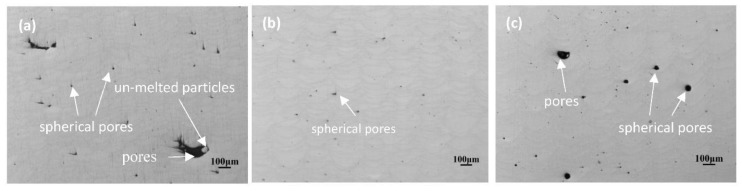
OM images of the cross-section microstructures of AlSi10Mg parts fabricated at different ET: (**a**) 100 μs; (**b**) 140 μs and (**c**) 180 μs. The PD is fixed at 80 μm.

In order to verify modelling results, corresponding experiments were carried out. The layered structures of the SLMed AlSi10Mg samples can be observed in Figure 10; furthermore, at the same PD value, we can also see that both the depth and the width of the melt pools both increased with an increasing ET. In Figure 10a, plenty of spherical gas pores (<20 μm) and an irregular lack of fusion pores (>100 μm) can be seen. These were induced by low input energy densities. The short liquid phase time and the high cooling rate of the melt pools reduced the viscosity of said melt pools (Figure 7a) and, as such, it was not possible to fully wet the powder particles around the melt pool. Therefore, some micropores and unmelted areas remained after alloy solidification, thus leading to a low relative density. When the ET was equal to 140 μs, the melt pool was in a good overlap due to large dimensions and flowability. As a result of longer liquid phase times and lower cooling rate, some bubbles escaped from the melt pool under Marangoni flow. Therefore, few gas pores (<20 μm) were observed (Figure 10b). However, when the laser ET reached 180 μs, the number of pores (<50 μm) in the melt pool began to increase (Figure 10c). Xiao et al. found that the gas solubility of the melt pool increased with increasing energy density during laser welds of the Al–Li alloy. In SLM, gas including evaporative magnesium [27], hydrogen [28], and argon will dissolve out but be detained in the melt pool under the condition of a low solidification rate; moreover, gas pores diffuse and expand easily, and larger keyhole pores are induced. Weingarten et al. [29] concluded that each time the following layer was scanned, the consolidated material was heat-treated if the penetration depth led to a more partial remelting at high energy density; as such, it is possible to conclude that trapped gas pores can enlarge and reduce the relative density. Besides this, a large of particles adhered to the melt pool or the evaporation of local material due to excessive laser energy input; as a result, a lack of powder and keyhole pores can also be caused in the neighbouring region.

**Figure 11:**
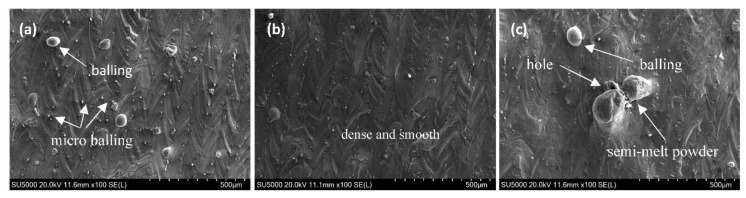
SEM images of the top surface morphologies of AlSi10Mg parts fabricated at different PD: (**a**) 60 μm; (**b**) 80 μm; and (**c**) 100 μm. The ET is fixed at 140 μs.

Figure 11 shows the top surface morphologies of AlSi10Mg parts produced by different PD values, with an ET fixed at 140 μs. It can be clearly seen that variation exists in these SEM images. As shown in Figure 11a, obvious “self–balling” can be seen at a PD of 60 μm. This was caused by the amount of energy density, which increased the liquid phase time and the maximum temperature of the melt pool. Here, an excessive liquid phase induced the formation of the balling phenomenon, with balling inevitably leading to pores. Besides, plenty of micrometre-scaled balls can be seen in between melt tracks. This was due to melt splashes from inside of the melt pool inevitably splattering on its edges under the action of the laser. When the PD was equal to 80 μm, a smooth surface was obtained (Figure 11b) and there were fewer pores and better properties in the cross section (Figure 10b and Figure 11b). However, when further increasing the PD to 100 μm, apparent balling occurred (Figure 11c). As previously stated, at a high PD value, the lower maximum temperature gave rise to small amounts of liquid formation, with higher cooling rates and a lower liquid lifetime and viscosity (Figure 6 and Figure 7). It can therefore be concluded that, the liquid metal transforms into near balling in a short period of time and will decrease the relative density and increase surface roughness. While increasing the laser power or decreasing the scanning speed, the same variation trend in respect of the balling phenomenon was found by Li et al. [8] and Han et al. [30].

In a track-by-track, layer-by-layer SLM process, the ability of metallurgical bonding plays an important role in the densification behaviour of the parts produced by SLM. Too much or too little energy input can cause serious pores and balling in parts, which ultimately degrades performance.

## 5. Conclusions

(1) As the laser moved from the centre of the first layer to the centre of the third layer, the maximum temperature of the melt pool decreased firstly and then increased slightly. The width and the depth of the melt pool increased continuously but in small amounts. This is mainly due to the heat accumulation phenomenon although the recoating phase.
(2) The cooling rate of the melt pool decreased from 7.93 × 10^6^ °C/s to 3.61 × 10^6^ °C/s as the ET increased from 100 μs to 180 μs. However, the rate rose significantly from 3.25 × 10^6^ °C/s to 7.48 × 10^6^ °C/s as the PD increased from 60 μm to 100 μm. The cooling rate during the recoating phase elevated as the ET increased and the PD decreased. This was due to increasing heat accumulation in the underlying solid material.
(3) The maximum temperature and the liquid lifetime rose as the ET increased and the PD decreased. However, the PD had more notable effects on the liquid lifetime.
(4) The dimensions of the melt pool increased (the width from 107.2 μm to 134 μm and the depth from 39.8 μm to 57.5 μm) as the ET elevated from 100 μs to 180 μs, however, said dimensions decreased (the width from 131.9 μm to 114 μm and the depth from 56.5 μm to 44.5 μm) as the PD elevated from 60 μm to 100 μm. It can be seen that the depth and the width were more sensitive to the PD than to the ET. The proper melt pool width (119.8 μm) and depth (48.65 μm) were obtained for a successful SLM process with a combination of ET = 140 μs and PD = 80 μm.
(5) The best forming quality—free of apparent pores, holes, cracks, and the balling phenomenon—was obtained at the optimised combination of ET = 140 μs and PD = 80 μm.

## Figures and Tables

**Table 1:** The process parameters in simulation and experiments.

Parameter	Value
Laser power, *P*	200 W
Hatching space, *S*	80 μm
Spot diameter, D	80 μm
Layer thickness	25 μm
Laser absorptivity of the Al powder, A	18% [13]
Spreading time	5 s
Exposure Time, ET	100, 120, 140, 160, 180 μs
Point Distance, PD	60, 70, 80, 90, 100 μm
